# Quality of Life Domains in Breast Cancer Survivors: The Relationship Between Importance and Satisfaction Ratings

**DOI:** 10.3389/fpsyg.2022.923537

**Published:** 2022-06-22

**Authors:** Andreas Hinz, Markus Zenger, Bjarne Schmalbach, Elmar Brähler, Dirk Hofmeister, Katja Petrowski

**Affiliations:** ^1^Department of Medical Psychology and Medical Sociology, University of Leipzig, Leipzig, Germany; ^2^Department of Applied Human Studies, University of Applied Sciences Magdeburg-Stendal, Stendal, Germany; ^3^Integrated Research and Treatment Center Adiposity Diseases, University of Leipzig, Leipzig, Germany; ^4^Department of Medical Psychology and Medical Sociology, University of Mainz, Mainz, Germany; ^5^Department of Psychosomatic Medicine and Psychotherapy, University of Mainz, Mainz, Germany

**Keywords:** quality of life, satisfaction, importance, breast cancer, psycho-oncology

## Abstract

**Objectives:**

Quality of life (QoL) has been the focus of increasing interest in oncology. QoL assessment instruments implicitly assume that each QoL domain has the same meaning for each patient. The objective of this study was to analyze the importance of and the satisfaction with QoL domains and to analyze the relationship between the two.

**Methods:**

A sample of 308 breast cancer survivors was examined twice with a three-month time interval. The women completed the two QoL questionnaires Questions of Life Satisfaction (FLZ-M), which measures participants' satisfaction with eight QoL domains and the subjective importance of those domains to them, and the EORTC QLQ-C30. A sample of 1,143 women from the general population served as controls.

**Results:**

Compared with the general population sample, the patients were less satisfied with their health and more satisfied with all other QoL domains. The subjective importance of health was lower in the patients' sample (Effect size: *d* = 0.38). Satisfaction with health and importance of health were slightly positively correlated (*r* between 0.05 and 0.08). The effect of QoL domain importance on general QoL was small (beta between −0.05 and 0.11), and interaction effects between domain importance and satisfaction on the prediction of global QoL were negligible.

**Conclusion:**

In addition to satisfaction with QoL dimensions, the subjective importance of these dimensions is relevant for psychooncological research and treatment. Health is not the only relevant QoL domain in breast cancer survivors, other domains such as finances also deserve health care providers' attention.

## Introduction

Quality of life (QoL) has gained increasing relevance in clinical research and practice. Breast cancer patients and survivors suffer from substantial detriments in QoL (Arndt et al., 2005; Mols et al., 2005; Arraras et al., 2018; Carreira et al., 2021). Though there is no generally accepted definition of QoL, most studies view QoL as a multidimensional construct composed of at least four domains: physical, mental, social, and autonomy. Multiple questionnaires that are either generic, e.g., SF-36 (Ware and Sherbourne, 1992), or disease-specific, e.g., EORTC QLQ-C30 (Aaronson et al., 1993), have been developed to measure QoL. In both cases, the participants are required to assess their degree of functioning or satisfaction with regard to certain QoL domains. These instruments do not however take into account the importance of the individual QoL domains to the respondents. Therefore, several approaches have been developed to include this issue, e.g., the Schedule for the Evaluation of Individual Quality of Life (SEIQoL) (Wettergren et al., 2009) and the Patient Generated Index (PGI) (Martin et al., 2007). These instruments allow the subjects to freely choose the domains they consider relevant for their QoL, a process that, on the one hand, captures useful data but on the other, comes at the cost of generating results that are difficult to compare. By contrast, the Fragebogen zur Lebenszufriedenheit (Questions on Life Satisfaction) FLZ-M (Henrich and Herschbach, 2000) is a questionnaire with a fixed set of QoL domains that measures both satisfaction and importance. The total QoL score of this questionnaire can be calculated as the weighted mean of the domain satisfaction scores, with the importance ratings as the weight factors. Several studies have shown that the weighting with importance ratings does not change the QoL total scores substantially (Rohrer and Schmukle, 2018). However, importance ratings are not only of interest as weighting factors, they are also of genuine interest. How is the subjective importance of a domain to a person associated with their satisfaction with that domain? Though one could assume that the importance of a domain (e.g., health) increases when problems in that domain occur, resulting in a negative correlation between importance and satisfaction, empirical studies failed to detect such negative correlations. Two studies with general population samples reported correlations between the importance of health and the satisfaction with health of *r* = 0.08 (Hinz et al., 2010) and *r* = 0.23 (Rohrer and Schmukle, 2018); a study with students found a correlation of *r* = 0.04 (Wu and Yao, 2006); and in a small sample of injection drug users, the correlation was *r* = 0.00 (Russell et al., 2006). A further issue is the contribution of single QoL domains to overall QoL scores. Here one can assume that subjectively important domains contribute more strongly to overall QoL assessments than less important domains. Rohrer and Schmukle (Rohrer and Schmukle, 2018) tested the “domain-importance-as-a-moderator” hypothesis, which states that those domains that are assessed to be subjectively important contribute more strongly to overall QoL scores than less important domains. In a large sample of the general population, they found that only five out of the 10 examined dimensions showed such a significant interaction effect; the effect was small (*B* = 0.02) and not statistically significant for the health domain.

In cancer research, such examinations of the relationship between subjective importance and satisfaction with life domains had yet to be performed; most studies that have focused on the relationship between importance and satisfaction have been performed in samples of the general population (Tiefenbach and Kohlbacher, 2015; Rohrer and Schmukle, 2018) or students (Wu and Yao, 2006; Wu, 2008a,b; Chen and Lin, 2014). For health care providers, however, it is important to understand what aspects of QoL their patients value highly, and how this subjectively attributed importance is related to overall satisfaction.

Summing up, the aims of this study were: (1) to compare the QoL (domain importance and domain satisfaction) of the cancer patients with the scores of the general population, (2) to analyze the relationship between importance and satisfaction ratings, and (3) to test the domain-importance-as-a-moderator hypothesis, analyzing the relationship between domain importance and the association between domain satisfaction and general QoL.

## Methods

### Sample of Breast Cancer Survivors

Study participants were German female breast cancer survivors undergoing routine radiologic after-treatment examinations in a radiologic practice. All of the women who came for medical appointments at the cooperating radiologic practice and had a breast cancer diagnosis (*n* = 358) were asked to take part in the study. Time since cancer diagnosis had to be at least 6 months; there were no further exclusion criteria. The first examination (t1) was performed at the radiologic practice during the women's clinical visits. Three months after t1, the participants were sent a letter with a questionnaire and a stamped return envelope (t2 examination). The study was performed between January 2012 and April 2013.

The aims and the content of the study were explained to the candidates, and all participating women provided informed consent. The Ethics Committee of the University of Leipzig approved the examination.

### Sample of the General Population

The reference data were taken from a nation-wide representative normative study (Daig et al., 2009) based on a representative sample of the German general population in terms of age and gender. Addresses were selected using the random-route technique. Of the 8,106 originally generated addresses, 5,036 individuals agreed to participate in the study (response rate: 62%). Informed consent was obtained from all participants. Further details of the sample are given elsewhere (Daig et al., 2009). From the sample, we selected a subsample (*n* = 1,143) of women by successively removing younger female patients, so that the age distribution matched the distribution of the patients' sample, arriving at a mean age of 66.2 ± 9.0 years.

### Instruments

#### FLZ-M

Participants' valuation of and satisfaction with life domains were assessed with the Fragebogen zur Lebenszufriedenheit (Questions on Life Satisfaction) FLZ-M (Henrich and Herschbach, 2000) at baseline (t1) and at follow-up (t2). The questionnaire covers eight domains of life that are assumed to be relevant to most adult people: friends/acquaintances, leisure activities/hobbies, health, income/financial security, work/profession, housing situation, family life/children, and partnership/sexuality. For each dimension, the respondent is asked to assess its importance to them (1 = not important, …, 5 = extremely important) and how satisfied they are with it (1 = dissatisfied, …, 5 = very satisfied). A sum score can be calculated by summing up the satisfaction scores, either unweighted or weighted with the subjective importance ratings. The FLZ-M has been applied in multiple medical fields, e.g., cancer (Sehlen et al., 2012; Amler et al., 2015), transplantation medicine (Baranyi et al., 2013; Benzing et al., 2016), surgery (Kovacs et al., 2011), orthopedics (Minzlaff et al., 2018), dermatology (Gieler et al., 2017), and psychiatry (Kröger et al., 2015). Normative values of the FLZ-M are available (Daig et al., 2009).

#### EORTC QLQ-C30

The QoL questionnaire EORTC QLQ-C30 (Aaronson et al., 1993) consists of 30 items and comprises five functioning scales (physical, role, emotional, social, and cognitive functioning), three symptom scales (fatigue, pain, and nausea/vomiting), six single-item scales, and a two-item global health/QoL scale. One of the two global health/QoL items is the question “How would you rate your overall QoL during the past week?” with seven answer options, ranging from 1 (very poor) to 7 (excellent). This item was used as the dependent variable in the regression analyses.

### Statistical Analysis

Mean score differences were tested with *t*-tests. The associations between importance and satisfaction ratings as well as the test-retest correlations were calculated with Pearson correlations. Effect sizes *d* were calculated according to Cohen (Cohen, 1988), relating the mean score differences to the pooled standard deviations. The domain-importance-as-a-moderator hypothesis was tested with regression analyses. The dependent variable of these analyses was the QoL item from the EORTC QLQ-C30; the independent variables were domain satisfaction, domain importance, and the interaction between satisfaction and importance. For the definition of this interaction, the importance was dichotomized so that both resulting subgroups had nearly equal sizes. The group with the lower importance ratings was coded with 0, and the group with the higher importance ratings with 1. The interaction was defined as the product of this dichotomized importance rating and the satisfaction rating. All statistics were performed with SPSS, version 25.

## Results

### Sociodemographic and Clinical Characteristics

Of the 358 women who were asked to take part in the study, 338 (94%) agreed to participate and completed the t1 questionnaire. After 3 months, 308 (91% of the 338 women) sent the t2 questionnaire back (86% of the eligible patients). The following analyses are restricted to those 308 women who took part both at t1 and t2. Their mean age was 66.1 ± 9.6 years. Further characteristics of the cancer survivors' sample are given in Table 1.

**Table 1:** Sociodemographic characteristics of the sample of cancer patients (*n* = 308) and the general population sample (*n* = 1143).

	**Patients**		**General population**	
	***n***	**%**	***n***	**%**
Age				
≤59 years	68	22.1	269	23.5
60–69 years	107	34.7	409	35.8
≥70 years	133	43.2	465	40.7
Civil status				
Living without partner	105	34.1	450	39.4
Living with partner	202	65.6	693	60.6
Missing	1	0.3	0	0
Education				
≤9 years	89	28.9	551	48.2
10–11 years	146	47.4	432	37.8
≥12 years	71	23.1	160	14.0
Missing	2	0.6	0	0
Time since diagnosis				
≤5 years	165	53.6		
>5 years	143	46.4		
Chemotherapy				
No	157	51.0		
Yes	151	49.0		
Radiotherapy				
No	50	16.2		
Yes	258	83.8		
Hormone therapy				
No	143	46.4		
Yes	158	51.3		
Missing	7	2.3		

### Importance and Satisfaction Mean Scores

Domain importance and satisfaction mean scores are given in Table 2. First, we consider the patients' t1 mean scores in comparison with the general population. Concerning domain importance ratings, the mean scores of the patients at t1 (as compared with the general population) were somewhat lower in the health domain and higher in the work and family life domains. Concerning satisfaction, the patients were slightly more satisfied than the general population in all dimensions with the exception of health, with which the patients were slightly (insignificant) less satisfied. When comparing the t1 baseline values with the t2 follow-up means, there were only very slight differences in the importance ratings as well as in the satisfaction ratings.

**Table 2:** Importance and satisfaction ratings (range: 1–5).

	**General population**		**Patients t1**		**Patients t2**		**Comparisons**			
							**t1-GP**		**t2-t1**	
	**M**	**SD**	**M**	**SD**	**M**	**SD**	**ES**	***p***	**ES**	***p***
Importance										
Friends	3.59	0.83	3.57	0.89	3.63	0.85	−0.02	0.712	0.07	0.127
Leisure time	3.20	0.96	3.31	0.89	3.29	0.84	0.12	0.070	−0.02	0.554
Health	4.62	0.60	4.39	0.61	4.49	0.58	–*0.38*	*<0.001*	*0.17*	*0.003*
Income	4.05	0.71	4.08	0.73	4.09	0.74	0.04	0.513	0.01	0.870
Work	3.15	1.38	4.02	0.75	4.10	0.71	*0.82*	*<0.001*	0.11	0.084
Housing	3.92	0.72	4.12	0.66	4.16	0.65	*0.29*	*<0.001*	0.06	0.389
Family life	4.09	0.89	4.43	0.72	4.44	0.67	*0.41*	*<0.001*	0.01	0.910
Partner	3.17	1.28	3.13	1.47	3.28	1.43	−0.04	0.638	0.12	0.127
Satisfaction										
Friends	3.86	0.79	4.01	0.90	4.04	0.80	*0.18*	*0.004*	0.04	0.436
Leisure time	3.64	0.85	3.76	0.90	3.74	0.85	*0.14*	*0.030*	−0.02	0.649
Health	3.44	0.95	3.36	1.07	3.35	1.06	−0.08	0.202	−0.01	0.714
Income	3.41	0.93	3.54	0.99	3.59	0.96	*0.14*	*0.032*	0.05	0.104
Work	3.36	1.03	3.57	0.96	3.63	0.94	*0.21*	*0.001*	0.06	0.147
Housing	4.01	0.79	4.28	0.74	4.31	0.71	*0.35*	*<0.001*	0.04	0.602
Family life	3.93	0.90	4.31	0.87	4.35	0.87	*0.43*	*<0.001*	0.05	0.511
Partner	3.27	1.18	3.59	1.27	3.61	1.28	*0.26*	*<0.001*	0.02	0.272

*M, mean; SD, standard deviation; ES, effect size; GP, general population; italics, statistically significant*.

### Associations Between Domain Importance and Domain Satisfaction, Temporal Stability

The correlations between importance and satisfaction ratings are presented in Table 3. With one exception (income; patients at t1), all correlations were positive in the general population and in the patient sample. While in some domains there were substantial positive associations with coefficients >0.40 in the patients' sample (domains: partner, leisure time, friends, family life), the correlations in the health domain were low, with coefficients of 0.07 (patients, t1), 0.05 (patients, t2), and 0.08 (general population).

**Table 3:** Relationship between importance and satisfaction; test-retest-correlations.

	***r*** **(Importance, Satisfaction)**						***r*** **(t1, t2) (Patients)**			
	**General population**		**Patients t1**		**Patients t2**		**Importance**		**Satisfaction**	
	***r***	***p***	***r***	***p***	***r***	***p***	***r***	***p***	***r***	***p***
Friends	*0.49*	*<0.001*	*0.45*	*<0.001*	*0.51*	*<0.001*	*0.67*	*<0.001*	*0.63*	*<0.001*
Leisure time	*0.53*	*<0.001*	*0.53*	*<0.001*	*0.40*	*<0.001*	*0.58*	*<0.001*	*0.49*	*<0.001*
Health	*0.08*	*0.009*	0.07	0.190	0.05	0.379	*0.58*	*<0.001*	*0.62*	*<0.001*
Income	*0.08*	*0.009*	−0.01	0.874	0.04	0.525	*0.54*	*<0.001*	*0.72*	*<0.001*
Work	*0.25*	*<0.001*	0.08	0.136	0.09	0.101	*0.50*	*<0.001*	*0.66*	*<0.001*
Housing	*0.41*	*<0.001*	*0.33*	*<0.001*	*0.32*	*<0.001*	*0.49*	*<0.001*	*0.59*	*<0.001*
Family life	*0.53*	*<0.001*	*0.43*	*<0.001*	*0.53*	*<0.001*	*0.59*	*<0.001*	*0.59*	*<0.001*
Partner	*0.55*	*<0.001*	*0.61*	*<0.001*	*0.66*	*<0.001*	*0.84*	*<0.001*	*0.73*	*<0.001*

*Italics, statistically significant*.

All temporal stability coefficients were between 0.49 and 0.84 (Table 3). On average, the importance ratings were not more and not less stable than satisfaction ratings. Health is in the middle range of the domains with regard to the temporal stability of importance and satisfaction (*r* = 0.58 and *r* = 0.62).

### Relationship Between Domain Importance, Domain Satisfaction, and General QoL

Table 4, upper part, presents the correlations between domain importance, satisfaction, and general QoL for t1. All satisfaction scores were positively associated with QoL; the highest association (*r* = 0.41) was found for health. For each domain, importance was also positively correlated with QoL, ranging from 0.02 to 0.20; however, two of these correlations (income and work) failed to reach statistical significance.

**Table 4:** Correlation and regression analyses, predicting global QoL from importance, satisfaction, and the interaction of importance and satisfaction, for patients at t1 and t2.

	**Correlations**				**Regression analysis**						
	***r*** **(Import., QoL)**		***r*** **(Satisfact., QoL)**		**Importance**		**Satisfaction**		**Interaction Imp**. *Satisf.		***R***
	***r***	***p***	***r***	***p***	**beta**	***p***	**beta**	***p***	**beta**	***p***	
Patients at t1											
Friends	*0.12*	*0.025*	*0.24*	*<0.001*	0.02	0.682	*0.21*	*0.021*	0.02	0.799	0.24
Leisure time	*0.20*	*<0.001*	*0.37*	*<0.001*	−0.02	0.688	*0.31*	*<0.001*	0.11	0.146	0.38
Health	*0.14*	*0.010*	*0.41*	*<0.001*	*0.10*	*0.041*	*0.34*	*<0.001*	0.08	0.249	0.43
Income	0.02	0.660	*0.26*	*<0.001*	0.03	0.612	*0.30*	*<0.001*	−0.05	0.486	0.27
Work	0.03	0.614	*0.32*	*<0.001*	0.00	0.985	*0.29*	*<0.001*	0.05	0.410	0.33
Housing	*0.14*	*0.011*	*0.30*	*<0.001*	0.05	0.395	*0.29*	*<0.001*	−0.01	0.848	0.30
Family life	*0.12*	*0.038*	*0.23*	*<0.001*	0.02	0.727	*0.18*	*0.019*	0.05	0.471	0.23
Partner	*0.14*	*0.010*	*0.29*	*<0.001*	0.02	0.736	*0.22*	*0.014*	0.07	0.355	0.29
Patients at t2											
Friends	*0.26*	*<0.001*	*0.42*	*<0.001*	0.11	0.065	0.14	0.149	*0.28*	*0.001*	0.45
Leisure time	*0.28*	*<0.001*	*0.45*	*<0.001*	0.10	0.060	*0.28*	*<0.001*	*0.18*	*0.013*	0.48
Health	*0.13*	*0.018*	*0.63*	*<0.001*	*0.10*	*0.020*	*0.63*	*<0.001*	−0.01	0.936	0.64
Income	0.09	0.099	*0.38*	*<0.001*	0.08	0.117	*0.34*	*<0.001*	0.06	0.381	0.39
Work	0.07	0.225	*0.38*	*<0.001*	0.04	0.484	*0.33*	*<0.001*	0.07	0.274	0.39
Housing	0.11	0.054	*0.26*	*<0.001*	0.02	0.782	*0.21*	*0.002*	0.09	0.180	0.27
Family life	0.05	0.346	*0.23*	*<0.001*	−0.05	0.421	*0.22*	*0.010*	0.06	0.441	0.24
Partner	0.10	0.078	*0.25*	*<0.001*	−0.02	0.785	0.16	0.112	0.15	0.075	0.27

*Italics, statistically significant*.

Similar results were obtained in the multiple regression analyses that included importance, satisfaction, and the importance ^*^ satisfaction interaction. The R coefficient in the right column in Table 4 denotes the multiple regression coefficient of the regression analysis. All domain satisfaction ratings significantly predicted QoL, with health receiving the highest beta score (0.34) of the eight domains. Health importance also contributed significantly to QoL (beta = 0.10, *p* = 0.041), while all other importance ratings failed to reach the significance level. There was no statistically significant interaction term for the t1 measurements. The beta coefficient of the interaction in the health domain (beta = 0.08) was positive and indicates, in line with the hypothesis, a slightly stronger association between satisfaction with a domain and QoL for those patients for whom health was relatively important compared with those for whom health was less important. However, the corresponding *p* value of 0.249 is far from being statistically significant.

The corresponding coefficients of the t2 measurement are given in the lower part of Table 4. Once more, health satisfaction was characterized by the strongest association with QoL in the univariate and the multivariate analyses. Two out of the eight interaction terms (friends and leisure time) became statistically significant; all other interactions failed to reach statistical significance.

## Discussion

Breast cancer survivors were more satisfied with their life than women from the general population with the exception of the health domain. The survivors' mean health satisfaction ratings were only slightly worse than those of the general population, possibly due to a response shift effect (Sprangers and Schwartz, 1999; Friedrich et al., 2019), or in other words, a recalibration of the health domain. Relatively high levels of general health satisfaction were also found in other studies with breast cancer survivors (Arndt et al., 2005). Here one has to take into account that such relatively positive assessments of general health can occur even when the patients are suffering from several symptoms and detriments to multiple special dimensions of QoL (Hinz et al., 2017).

Health is the life domain with the highest importance ratings in the general population, a finding that has also been found in other contexts (Hinz et al., 2010; Tiefenbach and Kohlbacher, 2015). Breast cancer survivors, however, attribute less importance to the health domain. This contradicts the popular assumption that life domains such as health gain in importance when problems in this domain occur. Our results show that areas other than health, e.g., work issues (Nilsson et al., 2016), are relevant for cancer survivors. The correlation between health importance and health satisfaction was slightly positive with coefficients of 0.08 (general population) and 0.07 (patients, t1), while a negative correlation (problems in a domain lead to a higher awareness and a higher importance rating) was expected. Such non-negative or positive correlations were also found in studies with the American general population (*r* = 0.23) (Rohrer and Schmukle, 2018) and HIV patients (*r* = 0.00) (Russell et al., 2006). This result, in accordance with the other positive correlations between importance and satisfaction ratings, underlines that health is not only relevant for people with health problems.

The domain-importance-as-a-moderator hypothesis postulates that among people who attribute a high importance to a domain, the association between their satisfaction with that domain and their overall QoL or life satisfaction is particularly high, at least higher than the association among people with low importance ratings. This hypothesis was not confirmed. Concerning health, both satisfaction with health (*r* = 0.41) and importance of health (*r* = 0.14) were positively correlated with QoL. The interaction term importance ^*^ satisfaction, however, had no significant predictive value for QoL. The coefficients were slightly positive (0.08) at t1 and nearly zero (-0.01) at t2. This means that the impact of health status on general QoL is not only relevant for survivors who are in poor health but also for those who rate their health as good, as well as those who do not attribute great importance to the health dimension in the direct importance rating.

We only considered patients' subjective importance ratings in our study. Physicians also have concepts about which QoL domains they think matter most to their patients. It has been shown that the assessment of anxiety, depression, and QoL in cancer patients markedly differs between patients and the physicians who are treating them (Singer et al., 2011; Fahsl et al., 2012), and as such, it is probable that such differences also occur in perceptions of the importance of QoL dimensions. A study with pediatric cancer survivors showed large differences in the importance assessments made by survivors, parents, and clinicians (Jones et al., 2018). Further research is warranted to study similarities and dissimilarities in the importance assessments of QoL domains in the context of shared decision making (Nakayama et al., 2020).

Some *limitations* of this study should be mentioned. Differences between the cancer sample and the general population sample with respect to time of survey and level of education (higher mean education level in the patients' sample) may have led to a certain bias. All assessments concerning importance and satisfaction were single-item questions according to the questionnaire. Scales with more items would probably have been more precise and reliable. However, there were no questionnaires available with scales measuring the importance of life domains. The interaction term importance ^*^ satisfaction was based on a dichotomized importance rating. The advantage of this procedure was clear interpretability; other calculations, e.g., product of the mean-centered importance and satisfaction scores would also be possible. The satisfaction ratings of the eight domains are intercorrelated, which led us to calculate only univariate ANOVAs. A multivariate analysis with all domains entering in the model to predict general QoL would have been possible in principle, however, the sample size was too small for a model with 8 ^*^ 3 = 24 interrelated predictors. In all of our statistical analyses, we considered the variables to be metric rather than ordinal. One can debate the metric nature of the variables. For the sake of uniform treatment and comparability with results in the literature, the statistical analyses here are limited to metric methods. The degree of generalizability of the findings obtained in this sample of breast cancer survivors to other groups of patients remains unclear. Our calculations of associations between importance and satisfaction were based on cross-sections data sets within t1 and within t2. A task for the future would be the analysis of associations between changes in importance and changes in satisfaction ratings to further elaborate their mutual relationships.

## Conclusion

In summary, the results show that other domains of QoL besides health are relevant for cancer survivors as well. Though the “domain-importance-as-a-moderator hypothesis” was not confirmed, the importance ratings provide relevant information for better understanding cancer survivors' QoL. Further research is needed to explore the association between importance and satisfaction including their changes.

## Data Availability Statement

The raw data supporting the conclusions of this article will be made available by the authors, without undue reservation.

## Ethics Statement

The studies involving human participants were reviewed and approved by Ethical Review Board of the University of Leipzig. The patients/participants provided their written informed consent to participate in this study.

## Author Contributions

AH designed the study. EB and MZ recruited patients and obtained data from the general population. DH, KP, and AH performed the statistical analyses. AH and BS wrote the first draft. AH and KP wrote the final version. All authors contributed to the article and approved the final version.

## Funding

The study was funded by the Deutsche Forschungsgemeinschaft, grant number HI 1108/5-1.

## Conflict of Interest

The authors declare that the research was conducted in the absence of any commercial or financial relationships that could be construed as a potential conflict of interest.

## Publisher's Note

All claims expressed in this article are solely those of the authors and do not necessarily represent those of their affiliated organizations, or those of the publisher, the editors and the reviewers. Any product that may be evaluated in this article, or claim that may be made by its manufacturer, is not guaranteed or endorsed by the publisher.
